# Performance of large language models in delivering accurate and comprehensible patient information on heart failure and cardiomyopathy

**DOI:** 10.3389/fdgth.2026.1847603

**Published:** 2026-06-09

**Authors:** Christoph Reich, Jule Leverenz, Charlotte Brand, Lasse Niemeier, Isabel Branzei, Mustafa Yildirim, Farbod Sedaghat-Hamedani, Ali Amr, Norbert Frey, Benjamin Meder

**Affiliations:** 1 Department of Internal Medicine III, Heidelberg Faculty of Medicine, Heidelberg University, Heidelberg, Germany; 2 Precision Digital Health Unit of the Department of Internal Medicine III, Heidelberg Faculty of Medicine, Heidelberg University, Heidelberg, Germany; 3 German Center for Cardiovascular Research (DZHK), Heidelberg, Germany

**Keywords:** artificial intelligence, cardiomyopathy, digital health, heart failure, patient education

## Abstract

**Background:**

Large language models (LLMs) are increasingly used by patients seeking cardiovascular health information through digital platforms. However, their accuracy and suitability for providing guidance on heterogeneous diseases such as cardiomyopathies and heart failure remain inadequately evaluated. This study systematically benchmarked state-of-the-art LLMs on patient-oriented heart failure and cardiomyopathy queries regarding clinical appropriateness and comprehensibility.

**Methods:**

Six prominent LLM Chatbots were tested on 50 expert-curated questions covering disease understanding and lifestyle advice. A web-based evaluation platform randomized and blinded responses for assessment by twelve reviewers (cardiologists, medical students, AI auto-graders). Responses were rated on a 1–5 Likert scale across nine domains, including appropriateness, readability, and empathy. Reviewers also chose their preferred model per question.

**Results:**

Linguistic complexity and output length varied substantially. Gemini provided the most readable responses (Flesch–Kincaid Grade 11.3 ± 1.9) but was among the most verbose (668.7 ± 116.1 words). Across 2,700 ratings, Gemini received the highest composite mean rating (4.41 ± 0.77), excelling in completeness and factual reliability, followed by Grok (4.23 ± 0.76). Confabulation avoidance scored consistently high across all models (4.49 ± 0.02), while conciseness scored lowest (3.81 ± 0.05). Consistently, evaluators selected Gemini as their preferred information source in 43.7%, followed by Grok (30.3%). Rating tendencies varied by evaluator group: Auto-graders gave the highest average scores (mean 4.58 ± 0.60), followed by students (4.10 ± 0.88), while experts were more conservative (3.79 ± 0.93).

**Discussion:**

All LLMs showed good accuracy avoiding medical misinformation, though variability exists in readability and comprehensiveness. While major factual errors or hallucinations were rare in our blinded evaluation, they were not entirely absent.

## Introduction

Heart failure affects millions of patients worldwide, representing a complex chronic condition that requires comprehensive patient education and ongoing self-management support. The paradigm of heart failure care has been shifting from a reactive, healthcare-focused model to a proactive, patient-centric approach supported by digital technologies (1). Effective patient information delivery has emerged as a critical component of heart failure care, with studies demonstrating that patients often feel inadequately informed about discharge information and lack readily available written materials to reinforce their learned knowledge (2). This transformation underscores the critical role of patient education at scale, as individuals empowered with knowledge are better equipped to monitor their condition, adhere to treatment, and make informed lifestyle decisions (3).

For the last decade, the internet has served as the primary, immediate source of health information. This trend is a core component of the broader E-health transformation, demonstrating the digital technologies' promise to reshape care delivery. European Union data from 2024 reflects this behavior: 58% of individuals surveyed accessed health information online, while significant proportions also booked appointments with a health practitioner (40%) or accessed personal health records (28%) (4). However, this prevalence of digital health seeking has a fundamental flaw: the overwhelming volume of search results often contains misinformation, leading to self-diagnosis errors, heightened health anxiety, or delayed professional care (5).

The recent emergence of large language models (LLMs) such as OpenAI's ChatGPT represents a revolutionary shift from static web pages to interactive, conversational information retrieval. These sophisticated artificial intelligence systems, trained on vast corpora of text, can generate nuanced, seemingly high-quality responses to complex user queries. Their potential to augment patient communication is significant; in a notable comparison of responses to patient questions on a public forum, evaluators preferred chatbot-generated answers to those of physicians, rating the AI's output as being of higher quality and more empathetic (6). This finding suggests that LLMs could become a powerful tool to help bridge the information gap in chronic disease management. The capabilities of LLMs clearly extend beyond patient education, demonstrating remarkable potential across various medical domains. For instance, recent studies show that models like GPT-4 can diagnose complex clinical cases with an accuracy that outperforms a majority of human clinicians, illustrating their power as a diagnostic support tool (7). However, the rapid integration of this technology into healthcare is accompanied by significant challenges. A primary concern is the reliability of the information provided. Systematic reviews of LLMs in cardiovascular medicine have found that while often accurate, their responses can also contain spurious misinformation, lack crucial context, or be based on outdated clinical guidelines (8, 9). Furthermore, LLMs are prone to “hallucination”—generating plausible-sounding but factually incorrect statements—as they are optimized for linguistic coherence rather than factual accuracy (10). These issues are compounded by the fact that LLM-generated text is often written at a high grade level, which can limit its accessibility for patients with lower health literacy (8).

As LLMs become more powerful and accessible, it is crucial to rigorously and systematically evaluate their performance, especially for patient-facing applications. While initial research has benchmarked individual models, often older versions of ChatGPT, direct, blinded comparisons of the newest generation of competing LLMs are lacking. The performance of state-of-the-art models from different developers in providing safe, accurate, and comprehensible information for specific, high-stakes conditions like heart failure and cardiomyopathies remains unclear. Thus, the objective of this study was to systematically benchmark the clinical performance and readability of six leading LLMs when generating responses to patient-oriented questions in this field and to critically evaluate their readiness for real-world application.

## Methods

### Study design

**Figure 1:**
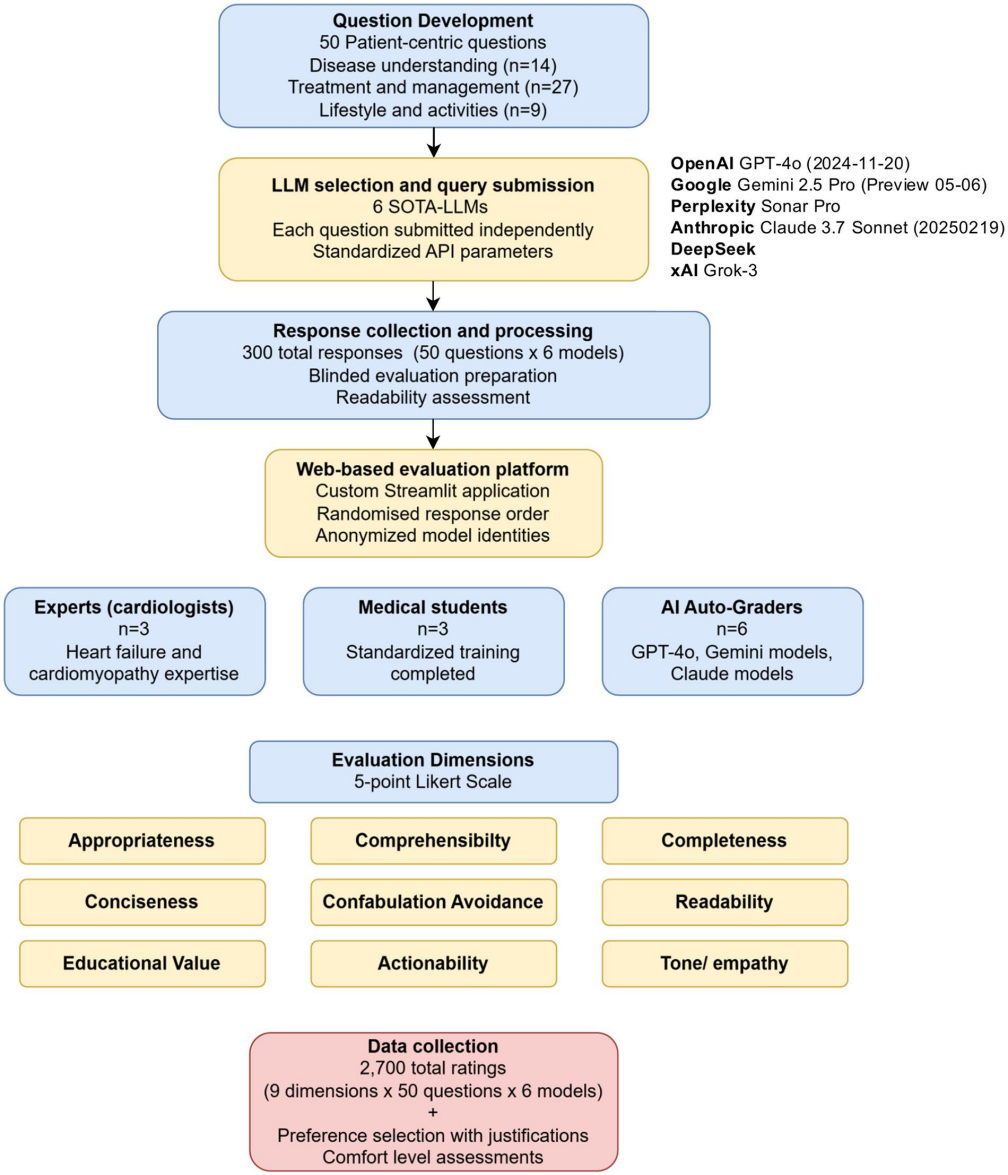
Study design and evaluation methodology. Flowchart illustrating the systematic, blinded, and multi-rater evaluation platform used to assess six SOTA Large Language Models. The study commenced with the development of 50 patient-centric questions categorized into three clinical topics. Responses (*n* = 300 total) were collected from the LLMs using standardized API parameters and evaluated on a custom web-based platform. Evaluation involved a panel of six human experts (three cardiologists and three medical students) and six AI Auto-Graders across nine distinct dimensions using a 5-point Likert scale.

This prospective evaluation study examined the quality of responses produced by six commonly used LLMs in response to patient-related questions about cardiomyopathies and heart failure. The study was designed to simulate real-world scenarios in which patients seek information, with expert clinical evaluation of the model's outputs across multiple quality dimensions and was conducted between February and June 2025. Six state-of-the-art (SOTA) LLM-based conversational agents were selected based on their public accessibility via chatbots and widespread use among patients seeking online medical information: OpenAI GPT-4o (2024-11-20), DeepSeek Chat, Gemini 2.5 Pro (Preview 05-06), Anthropic Claude 3.7 Sonnet (20250219), Perplexity Sonar Pro, and xAI Grok-3 (Figure 1).

### Question development and response collection protocol

A comprehensive set of 50 patient-centric questions was systematically developed focusing on core domains relevant to cardiomyopathy and heart failure management (Table 1). Questions were categorized into disease understanding and diagnosis (14 questions), treatment and management (27 questions), and lifestyle and daily activities (9 questions). The questions were derived from common consultations in clinical practice, frequently asked questions on patient forums including Reddit and Quora, and current medical literature and evidence-based guidelines. The final question set was reviewed for clinical relevance and patient-centered focus. Each question was then submitted individually and identically to all six LLMs using their respective API interfaces. To ensure response independence and prevent context carryover effects, each query was posed as a fresh prompt without prior conversation history. No prompt engineering strategies were applied: each question was submitted zero-shot as a single user message, with no system prompt, role priming, few-shot examples, chain-of-thought instructions, or output formatting or length specifications. All responses were collected under controlled conditions with standardized parameters where applicable. All model outputs were converted from markdown directly except for Perplexity responses, where references and citations were removed in a preprocessing step to maintain evaluator blinding, as these distinctive features would have otherwise made the model identity apparent. Response readability was quantitatively assessed using validated metrics including the Flesch–Kincaid Grade Level, which indicates the U.S. education level required to comprehend the text. Word count was recorded as a measure of response verbosity.

**Table 1:** Fifty study questions sent to LLM-APIs for evaluation.

Question
**Disease Understanding & Diagnosis (*n*** **=** **14)**
What is heart failure?
What's my ejection fraction, and what does it mean?
I was diagnosed with heart failure. What are the most common symptoms I should expect?
What tests are needed to diagnose cardiomyopathy?
How can genetic testing help in cardiomyopathy?
I have a heart failure diagnosis, but my ejection fraction is preserved. Why is that?
Is cardiomyopathy reversible?
What is sudden cardiac death risk in hypertrophic cardiomyopathy?
Will my heart function improve over time, or will it get worse?
I gained five kilos in the last two weeks and have edema in both legs. What should I do?
What caused my heart failure? Is it genetic?
My father has a defibrillator and a thick heart. His mother died suddenly when he was young. I play on an amateur football team and I don't have any symptoms. Should I be worried?
Heart disease runs in my family – my dad had dilated cardiomyopathy. Should I get tested or screened for it, even if I have no symptoms?
What is hypertrophic cardiomyopathy?
**Treatment & Management (*n*** **=** **27)**
I was diagnosed with heart failure and EF is 30%. I was prescribed a lot of medication. Do I need to take all of them?
I have dilated cardiomyopathy and experienced cardiac decompensation 3 months ago. Should I get vaccinated?
What are the main medications for heart failure and why are they important?
Three years ago, I was diagnosed with DCM. Now, I am experiencing palpitations, and my smartwatch is registering high heart rates. What should I do?
My heart has improved over the last few years, and my doctor told me that I now have nearly a normal EF. Does that mean I can stop taking my heart medication?
I have obstructive hypertrophic cardiomyopathy. Will I need to undergo cardiac surgery like my mother did?
I take Entresto (24/26 mg) twice daily and my blood pressure is 130/80 mmHg. Can I stop taking it?
I missed my heart failure medication yesterday. Should I go to the doctor?
I have had a persistent cough since I was diagnosed with heart failure. Could it be a side effect of my medication or the heart failure itself?
I have dilated cardiomyopathy and have been prescribed heart failure medication. What are the goals of my treatment plan?
Am I taking too many drugs for my heart failure? I take bisoprolol, empagliflozin, Entresto, and eplerenone. Can I stop taking some of them?
I am a 40-year-old with HCM and atrial fibrillation. I underwent catheter ablation for my atrial fibrillation last year, and I no longer experience any symptoms. Can I stop taking blood thinners?
My doctor told me that I need a myocardial biopsy due to my reduced EF and arrhythmia. Why is it necessary, and what are the risks?
How should my blood pressure and diabetes be managed with heart failure?
Should I use wearable devices, such as smartwatches or patches, for my DCM diagnosis?
What if my genetic test is positive? What does that mean for me and my family?
What if my genetic test is negative? Does it mean my cardiomyopathy isn't genetic?
Can cardiomyopathy get better or worse over time? What is my prognosis?
What is an ICD and how does it help?
Could I ever need a heart transplant because of my cardiomyopathy?
Is pregnancy safe if I have cardiomyopathy?
I am pregnant and have dilated cardiomyopathy. I need to know if I need to have a C-section.
My symptoms have clearly improved over the last year since I started taking mavacamten for oHCM. My doctor told me that I still have normal heart function. Can I stop taking mavacamten now?
If I feel fine, do I still need treatment or follow-up for cardiomyopathy?
I have HCM and atrial fibrillation with a fast heart rate. Should I undergo cardioversion?
What is TASH, and what are its risks?
Is it normal to need multiple carpal tunnel surgeries when I also have HCM?
**Lifestyle & Daily Activities (*n*** **=** **9)**
Can I travel with heart failure, and what precautions should I take?
Is it safe to exercise when being diagnosed with dilated cardiomyopathy?
I have the same HCM variant as my father, but I have not yet shown any signs of heart disease. Are there any exercise restrictions for me since I compete in sports?
What is the impact of my condition on sexual activity? Are there any precautions I need to take?
I have an implanted cardioverter-defibrillator and I live in Germany. Are there any limitations on my ability to drive?
I have dilated cardiomyopathy. What lifestyle changes should I make?
I have DCM. How much fluid should I drink each day? How should I monitor my fluid intake and output?
Can I drink alcohol with cardiomyopathy?
Can I drink caffeine with cardiomyopathy?

Questions were sourced from clinical practice observations, patient forum queries (Reddit, Quora), evidence-based guidelines and medical literature review. Questions were categorized by topic: Disease Understanding & Diagnosis (*n* = 14), Treatment & Management (*n* = 27), Lifestyle & Daily Activities (*n* = 9), with a patient-centered focus, comprehensive topic coverage, and standardized formatting.

### Evaluation platform and blinding

A custom web-based evaluation platform was developed using the Streamlit framework to facilitate blinded evaluation (*LLM Response Evaluator*, available at https://github.com/q050cr/llm_grading_app). The platform anonymized model identities, randomized response order for each question-evaluator pair, and provided structured data collection tools with built-in validation checks. Each response was evaluated independently by three cardiologists with expertise in heart failure and cardiomyopathies and three student graders for the main analysis. To perform a more objective sensitivity analysis and evaluate the consistency and scalability of the evaluation process, we conducted an analysis with six AI-based graders (6 automated evaluation systems using function calling from OpenAI GPT-4o, Google Gemini 2.0 Flash, Google Gemini 2.5 Pro, Anthropic Claude Sonnet 3.5 and Sonnet 4; detailed description in Supplementary Methods). The six automated graders were drawn from three independent provider companies (2 × OpenAI, 2 × Anthropic, 2 × Google) as a deliberate design choice to mitigate potential same-company grading bias; all summary statistics pool these six auto-graders with six human raters. We implemented these systems via structured function-calling prompts. Each system evaluated the same responses independently using the identical nine-dimension rubric. All human evaluators received standardized instructions and completed practice evaluations before beginning the formal assessment. Each response was evaluated across nine predefined dimensions using a 5-point Likert scale, where 1 represented very poor performance and 5 represented excellent performance. The dimensions assessed were appropriateness (clinical relevance and accuracy in addressing the specific question), comprehensibility (clarity and ease of understanding for a lay audience), completeness (inclusion of all relevant clinical aspects), conciseness (avoidance of unnecessary repetition or excessive verbosity), confabulation avoidance (freedom from fabricated or inaccurate information), readability (overall structure and linguistic clarity), educational value (informativeness and learning potential), actionability (presence of clear practical recommendations), and tone/empathy (appropriateness of emotional tone and patient sensitivity). For each question, evaluators selected the single best response and provided justification using a standardized checklist. Evaluators also indicated their comfort level with patients relying on each preferred response using a three-point scale (*fully comfortable, somewhat comfortable, or not comfortable*).

### Statistical analysis

Statistical Analysis Data were collected from human raters using a 5-point Likert scale across nine evaluation dimensions, alongside calculated continuous metrics such as response word count and Flesch–Kincaid Grade Level (FKGL). Descriptive statistics, including means, standard deviations (SD), and 95% confidence intervals (CI), were calculated for each model and evaluation dimension. Overall model performance and ranking were determined based on the mean composite score derived from all ratings and stratified by rater type. Qualitative preference data (model choice and rationales) were summarized using frequencies and percentages. The relationship between automated AI scores and human consensus ratings was examined to validate the automated grading system. All analyses were performed using Python 3.11 (Python Software Foundation, Wilmington, DE) and R version 4.3.0 (R Foundation for Statistical Computing, Vienna, Austria).

## Results

### LLM responses vary not just in length, but also in how easy they are to understand

**Figure 2:**
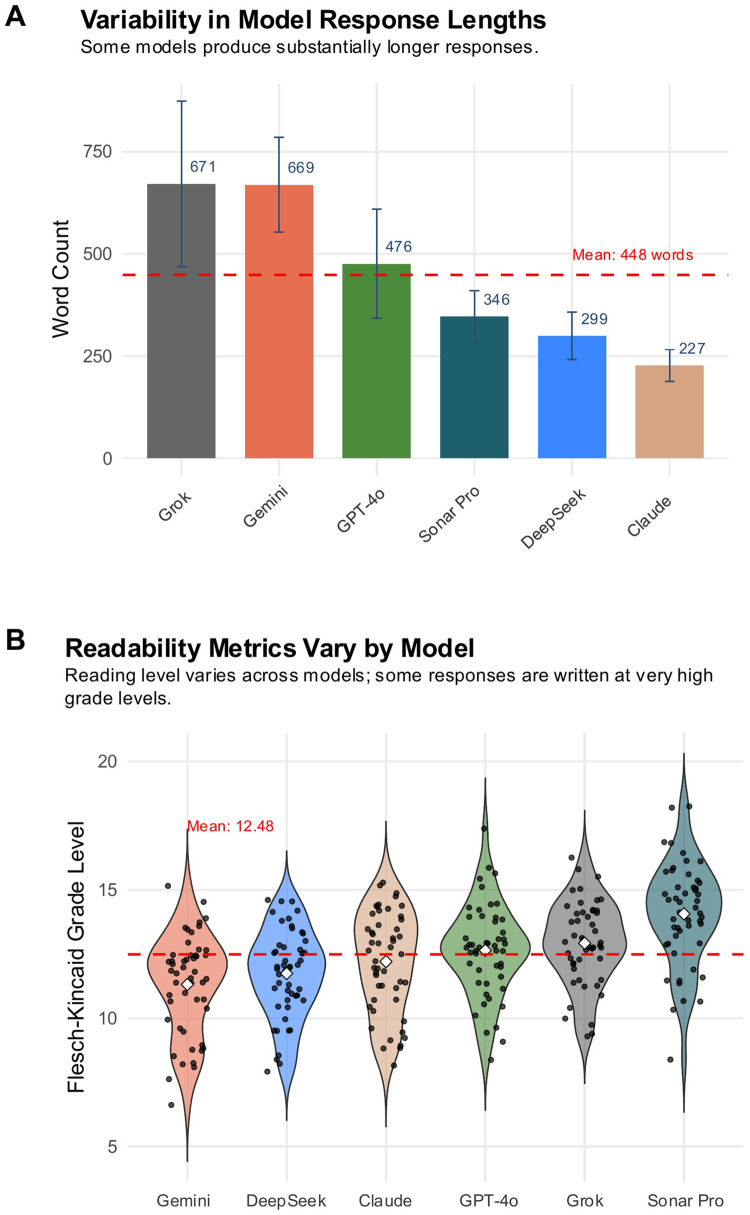
Variability in large language model response length and linguistic complexity. **(A)** Distribution of response word counts across the six LLMs. Data are presented as mean word count with standard deviation (error bars). The mean word count across all models was 448 words. Grok and Gemini generated the longest responses (671 and 669 words, respectively), while Claude produced the most concise (227 words). **(B)** Distribution of the Flesch Reading Ease score for responses. The score indicates linguistic complexity, with a higher number corresponding to easier readability. The mean Flesch–Kincaid Grade Level across all models was 12.48, substantially exceeding recommended guidelines (6th–8th grade) for patient-facing materials and potentially limiting accessibility.

Substantial variation was observed in linguistic complexity and response length across the six LLMs (Figures 2A,B). Response word counts ranged from Claude's most concise outputs (226.9 ± 38.9 words) to the most verbose responses from Gemini (668.7 ± 116.1 words) and Grok (671.2 ± 202.3 words). GPT-4o generated responses of intermediate length (475.8 ± 133.4 words), while DeepSeek (299.5 ± 57.8 words) and Sonar Pro (346.4 ± 63.4 words) produced moderately sized responses. Readability metrics revealed marked differences in linguistic complexity across models (ANOVA *p*-value < 0.001), consistent across all question categories - including disease understanding and diagnosis, treatment and diagnosis, and lifestyle and daily activity (see Supplementary Figure S1). Gemini provided the most accessible content with a Flesch–Kincaid Grade Level (FKGL) of 11.3 ± 1.9, indicating content suitable for high school-level comprehension. In contrast, Perplexity generated the most complex responses with a FKGL of 14.1 ± 2.0. Claude, GPT-4o, and Grok demonstrated moderate complexity levels with FKGLs of 12.2 ± 1.9, 12.6 ± 1.8, and 12.9 ± 1.6, respectively. Further details on readability and word count are given in Supplementary Tables S1, S2.

### Clarity, completeness, and clinical appropriateness drive top model rankings

**Figure 3:**
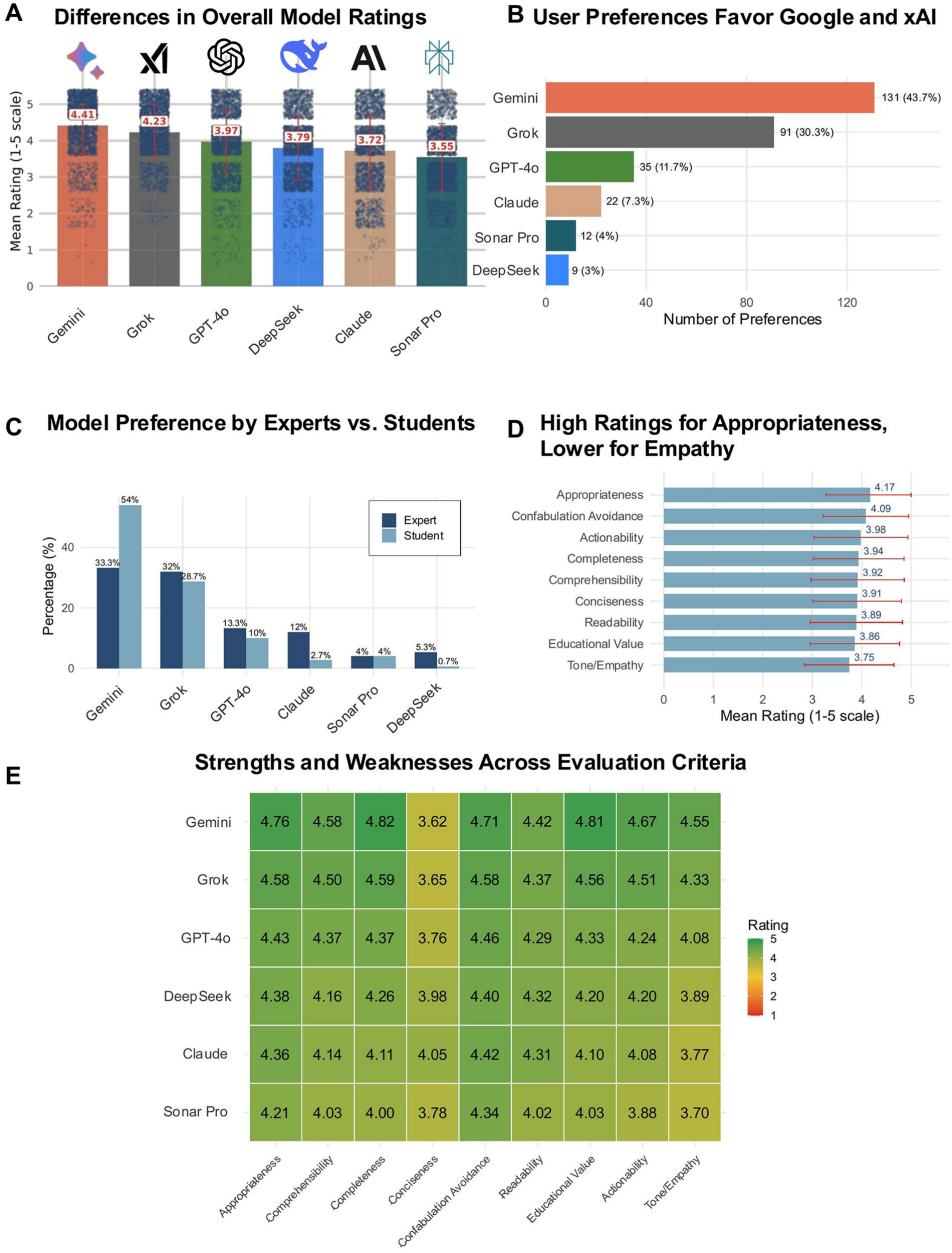
Overall performance ratings and rater preferences. **(A)** Differences in mean overall composite rating (1–5 scale) for each LLM based on assessment by human evaluators. Data points represent individual ratings. **(B)** Percentage and count of human evaluator preferences for the best model response across the 300 total evaluations. Gemini was selected most frequently (43.7%), followed by Grok (30.3%) and GPT-4o (11.7%), with lower preference rates observed for Claude, Sonar Pro, and DeepSeek. **(C)** Stacked bar chart illustrating the proportion of total preferences (*n* = 300) received by each LLM, stratified by the rater group (Cardiologists vs. Medical Students). **(D)** Bar plot illustrating the strengths and weaknesses of each LLM across the nine evaluation criteria. Scores range from 1 to 5, with higher scores indicating better performance. **(E)** Heatmap comparison of LLM performance across nine evaluation domains. Gemini performs strong overall, but no model dominates all categories. Conciseness is a consistent weakness across models.

Across 2,700 total ratings (9 dimensions × 50 queries × 6 raters), Gemini demonstrated the highest overall performance, with a mean composite score of 4.41 ± 0.77 (SD) on a 5-point scale as assessed by the human evaluators. This was followed by Grok (4.23 ± 0.76), GPT-4o (3.97 ± 0.87), DeepSeek (3.79 ± 0.88), Claude (3.72 ± 0.99), and Sonar Pro (3.55 ± 0.92) (Figure 3A). Evaluator preferences strongly aligned with quantitative ratings: Gemini was selected as the preferred model in 131 of 300 evaluations (43.7%), followed by Grok in 91 evaluations (30.3%), and GPT-4o in 35 evaluations (11.7%) (Figure 3B). Claude, Sonar Pro, and DeepSeek received notably fewer preferences at 22 (7.3%), 12 (4.0%), and 9 (3.0%) evaluations, respectively. Qualitative rationales for human model preference further supported these rankings and showed that the most frequently cited reasons for choosing Gemini included clearer explanations (35.1%), more complete content (31.3%), and better alignment with clinical practice (13.7%) (Supplementary Table S3). Grok was often rated for its ease of understanding (31.9%), clarity (26.4%), and completeness of content (20.9%). GPT-4o was recognized for its simplicity (31.4%) and clarity (25.7%), though not as much for its alignment with clinical practice or completeness. Models with lower preference rates, such as Sonar Pro and DeepSeek, received limited recognition across all qualitative dimensions. Individual rater-level preferences are reported in Supplementary Table S4, confirming that trends were robust across individuals and not driven by outliers.

### Automated grading confirms human evaluation patterns

**Figure 4:**
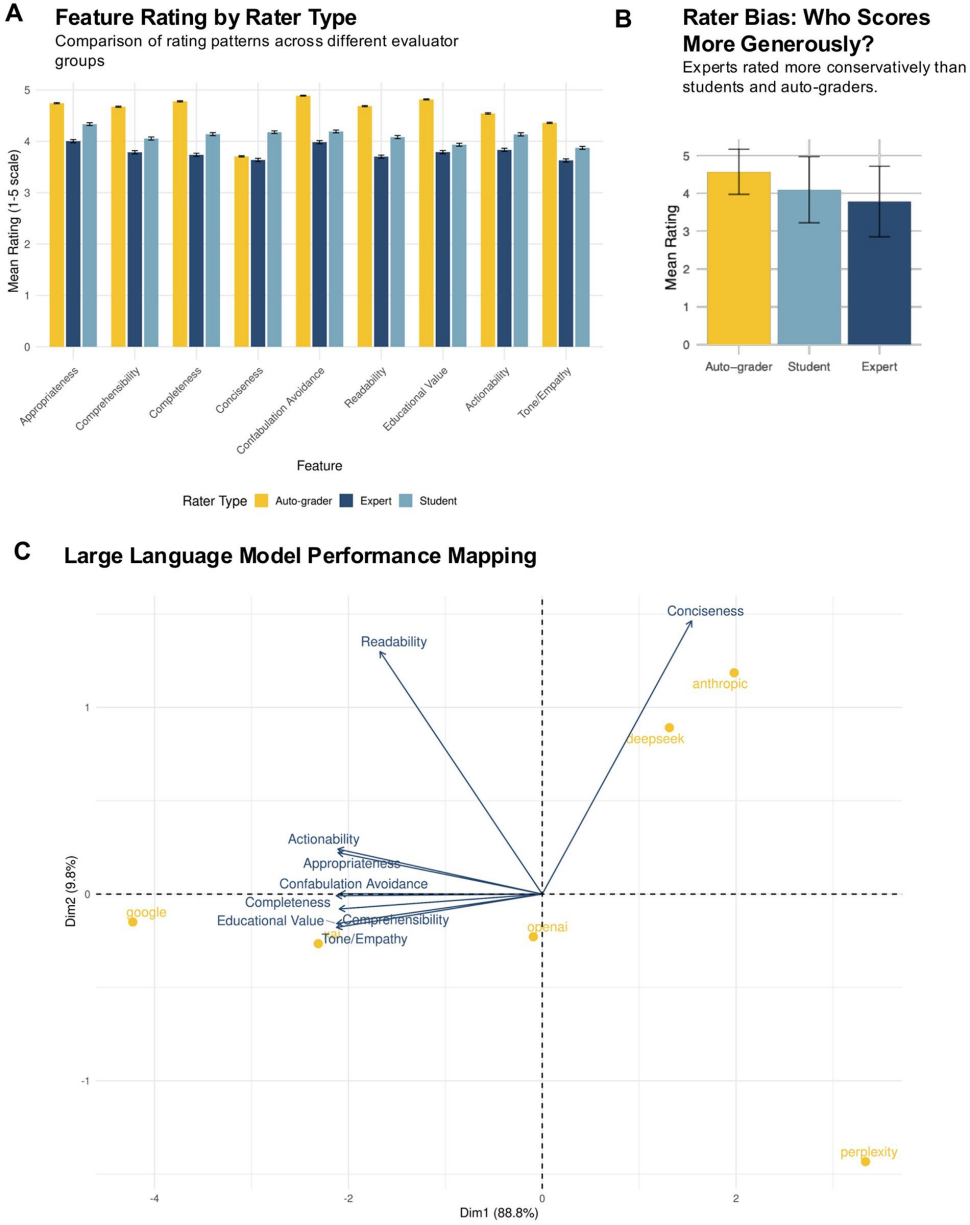
Multivariate patterns and rater bias. **(A)** Bar plot comparing the mean rating for each of the nine features across the three rater groups: Auto-grader, Student, and Expert. The plot illustrates that Auto-graders generally assigned the highest scores, while Expert Cardiologists assigned the most conservative scores across all features. **(B)** Bar plot summarizing the mean overall composite rating across the three evaluator types: Auto-grader, Student, and Expert. Experts rated more conservatively (lowest mean score) than students and auto-graders. **(C)** Principal Component Analysis plot showing the relationship between evaluation features (vectors) and the LLMs (points). The primary axis, Dimension 1 (Dim1), explained 88.8% of the variance and represents a trade-off between Completeness/Educational Value and Conciseness. The secondary axis, Dimension 2 (Dim2), explained 9.8% of the variance and is primarily related to Readability. Gemini and Grok cluster strongly with completeness and educational value.

Autograders are based on LLMs and used to evaluate texts from different sources with defined criteria. Interestingly, the aggregate autograder scores for the here tested LLMs closely mirrored the human rankings. Gemini achieved the highest mean composite score (4.68 ± 0.56), followed by DeepSeek (4.60 ± 0.57), xAI (4.59 ± 0.61), Anthropic (4.58 ± 0.55), OpenAI (4.55 ± 0.66), and Perplexity (4.46 ± 0.60) (Supplementary Figure S2A). Compared to human graders, AI-based scores were generally higher and less variable, likely reflecting the consistent application of scoring criteria across prompts (Figures 4A,B). The combined human–AI composite analysis (*n* = 5,400 ratings per model) preserved the relative ranking of the top-performing models (Gemini, Grok, and GPT-4o) while reducing variability among models (see Supplementary Figure S3). Notably, although AI graders tended to assign higher and more consistent ratings overall (Figure 4C), they still identified low-quality outputs. Only xAI, DeepSeek, and OpenAI ever received minimum scores of 1, whereas Gemini and Claude were never assigned the lowest score. A *post-hoc* same-company bias analysis found no statistically significant own-company grading effect for any auto-grader family after accounting for model quality differences and scale-ceiling effects (Supplementary Methods; Supplementary Table S5).

### Analysis of low-quality outputs and critical failures

**Figure 5:**
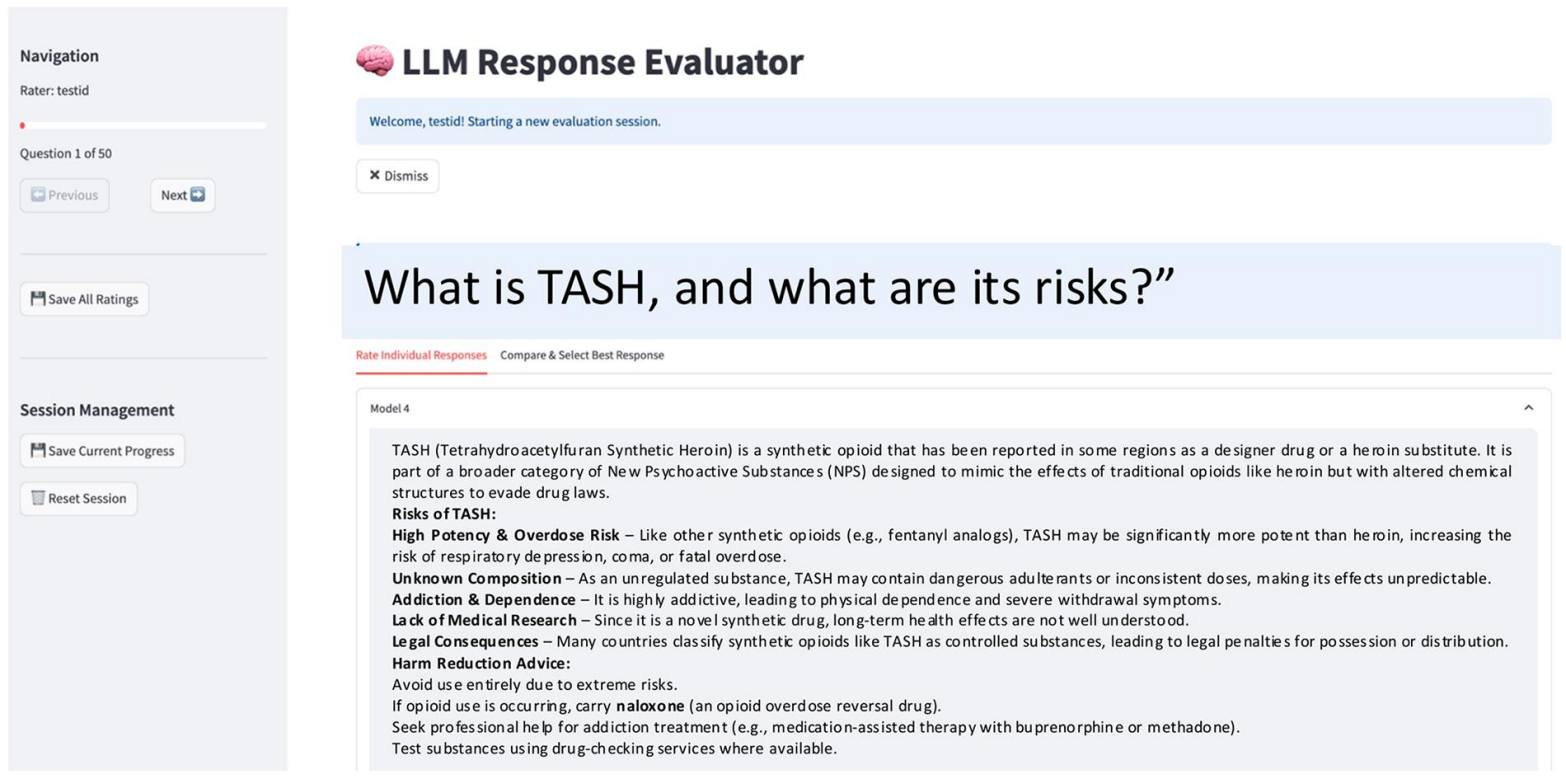
Example of hallucination in response to an ambiguous query. Screenshot of the evaluation platform showing a representative LLM response to the ambiguous query. The LLM hallucinates a confident, detailed, medically-relevant definition for a synthetic opioid, TASH (Tetrahydroacetylfuran Synthetic Heroin), along with its risks and harm reduction advice.

Closer analysis of low ratings (scores of 1 or 2, which indicate significant errors or unsuitability) revealed systematic differences in failure rates. Human evaluators gave low ratings (1, 2) most frequently to Claude (13.4%) and Sonar Pro (12.3%), and less often to other models (2.2%–6.0%) (Table 2). However, every model, including top-ranked Gemini and Grok, exhibited occasional hallucinations when responding to more ambiguous or obscure queries. A striking example of this failure mode was observed in the query, “What is TASH, and what are its risks?” (Figure 5). In the absence of clear context (TASH: Transcoronary Ablation of Septal Hypertrophy), one model generated a fully fabricated drug entity a synthetic opioid with a detailed risk profile and harm-reduction guidance rather than recognising TASH as a cardiac procedure, while other models identified plausible (if occasionally incorrect) medical meanings of the acronym. Further failure types included (1) Missed clinical urgency with a potentially harmful recommendation: in response to sudden 5-kg weight gain and bilateral leg oedema, one model listed renal and hepatic causes and advised the patient to “stay well-hydrated by drinking plenty of water” - the opposite of the recommended fluid restriction (≤1.5 L/day) in heart failure. (2) Potentially harmful framing: asked whether to vaccinate after recent cardiac decompensation from dilated cardiomyopathy, one model led with two case reports of LVAD-requiring vaccine-induced myocarditis before mentioning the strong guideline recommendation to vaccinate. (3) Missed diagnostic signal: asked whether repeated carpal tunnel surgeries are normal in the context of HCM, one model stated that no connection between the two conditions exists and offered only generic surgical advice, missing the recognised association with ATTR cardiac amyloidosis. Together, these results illustrate a spectrum of clinically relevant failure modes, including contextual misinterpretation, omission of critical diagnostic cues, and, in rare cases, the generation of entirely fabricated medical entities presented with unwarranted clinical confidence.

**Table 2:** Distribution of low-quality response ratings (scores of 1 or 2) by large language model.

Model	Low ratings (1-2)	Low rating %
Claude	362	13.4%
Sonar Pro	331	12.3%
Deepseek	161	6.0%
GPT-4o	125	4.6%
Gemini	78	2.9%
Grok	60	2.2%

This table shows the frequency and percentage of responses receiving a low rating (indicating significant errors or unsuitability) across all human evaluators (*n* = 2,700 total ratings per model). The data highlights the differential failure rates across models, with Claude and Sonar Pro exhibiting the highest percentage of low ratings, and Grok demonstrating the lowest.

### Feature-level strengths and weaknesses across models

Analysis of individual evaluation features showed systematic differences (Table 3). Gemini consistently achieved the highest ratings for appropriateness (4.76, 95% CI 4.72–4.80), comprehensibility (4.58, 95% CI 4.53–4.62), completeness (4.82, 95% CI 4.78–4.85), and educational value (4.81, 95% CI 4.77–4.85). Grok also performed strongly across most dimensions, particularly completeness (4.59, 95% CI 4.54–4.64) and appropriateness (4.58, 95% CI 4.54–4.63). By contrast, Sonar Pro and Claude showed lower scores across most features, particularly tone/empathy (3.70 and 3.77, respectively). Conciseness was the weakest dimension overall, especially for Gemini (3.62, 95% CI 3.56–3.68) and Grok (3.65, 95% CI 3.60–3.71). Figures 3D,E illustrates these feature-level patterns. Table 4 highlights best- and worst-performing features per model. Principal component analysis revealed that nearly all variance (98.6%) in evaluation scores was explained by two axes: a dominant trade-off between completeness/educational value and conciseness (PC1, 88.8% of variance) and a secondary axis related to readability (PC2, 9.8%) (Figure 4C). Models such as Gemini and Grok aligned strongly with completeness and educational value, whereas Claude positioned close to conciseness.

**Table 3:** Mean performance ratings for each large language model across nine evaluation features (5-point scale).

Feature	Claude	DeepSeek	Gemini	GPT-4o	Sonar Pro	Grok
Appropriateness	4.36 (4.29–4.43)	4.38 (4.32–4.44)	4.76 (4.72–4.80)	4.43 (4.36–4.49)	4.21 (4.14–4.28)	4.58 (4.54–4.63)
Comprehensibility	4.14 (4.06–4.23)	4.16 (4.08–4.23)	4.58 (4.53–4.62)	4.37 (4.30–4.43)	4.03 (3.96–4.11)	4.50 (4.45–4.55)
Completeness	4.11 (4.04–4.19)	4.26 (4.19–4.33)	4.82 (4.78–4.85)	4.37 (4.30–4.43)	4.00 (3.93–4.08)	4.59 (4.54–4.64)
Conciseness	4.05 (3.99–4.11)	3.98 (3.92–4.04)	3.62 (3.56–3.68)	3.76 (3.70–3.81)	3.78 (3.72–3.84)	3.65 (3.60–3.71)
Confabulation Avoidance	4.42 (4.36–4.49)	4.40 (4.33–4.47)	4.71 (4.66–4.76)	4.46 (4.40–4.53)	4.34 (4.28–4.41)	4.58 (4.52–4.63)
Readability	4.31 (4.24–4.38)	4.32 (4.25–4.38)	4.42 (4.36–4.48)	4.29 (4.23–4.36)	4.02 (3.94–4.10)	4.37 (4.31–4.42)
Educational Value	4.10 (4.02–4.17)	4.20 (4.12–4.27)	4.81 (4.77–4.85)	4.33 (4.26–4.40)	4.03 (3.96–4.11)	4.56 (4.51–4.61)
Actionability	4.08 (4.00–4.15)	4.20 (4.13–4.27)	4.67 (4.62–4.71)	4.24 (4.18–4.31)	3.88 (3.80–3.95)	4.51 (4.46–4.56)
Tone/Empathy	3.77 (3.70–3.84)	3.89 (3.83–3.96)	4.55 (4.50–4.59)	4.08 (4.02–4.14)	3.70 (3.64–3.76)	4.33 (4.28–4.38)

The mean score and 95% confidence interval are reported for each of the nine performance dimensions, as assessed by the human evaluators. The CI provides the range within which the true mean rating is expected to fall 95% of the time. The results demonstrate Gemini's consistently high performance in features like Completeness, Appropriateness, and Educational Value, while all models generally scored low on Conciseness.

**Table 4:** Highest and lowest rated performance features for each large language model.

Model	Highest	Lowest
Claude	Confabulation Avoidance [4.42 (4.36–4.49)]	Tone/Empathy [3.77 (3.70–3.84)]
DeepSeek	Confabulation Avoidance [4.40 (4.33–4.47)]	Tone/Empathy [3.89 (3.83–3.96)]
Gemini	Completeness [4.82 (4.78–4.85)]	Conciseness [3.62 (3.56–3.68)]
GPT-4o	Confabulation Avoidance [4.46 (4.40–4.53)]	Conciseness [3.76 (3.70–3.81)]
Sonar Pro	Confabulation Avoidance [4.34 (4.28–4.41)]	Tone/Empathy [3.70 (3.64–3.76)]
Grok	Completeness [4.59 (4.54–4.64)]	Conciseness [3.65 (3.60–3.71)]

This summary table isolates the single best-performing and single worst-performing feature for each LLM based on the mean ratings from the human evaluators (data sourced from Table 3). The corresponding mean score and 95% confidence interval are included for context, highlighting the primary trade-offs in model design, particularly the consistent struggle with Conciseness versus the strengths in clinical-facing features like Confabulation Avoidance and Completeness.

## Discussion

In this systematic, blinded evaluation of six leading large language models, we benchmarked their performance in generating responses to patient-oriented questions on heart failure and cardiomyopathies. Our findings reveal a striking heterogeneity in quality, readability, and verbosity among these state-of-the-art systems. In general, the provided answers were considered well-structured and appropriate. However, our study also identified significant, persistent challenges across all models - including poor readability, a trade-off between completeness and conciseness, and critical gaps in contextual understanding and information currency - that temper enthusiasm for their immediate, unsupervised use in patient-facing applications.

The preference for Gemini and Grok was driven by their higher scores in completeness and educational value, as validated by both human and AI-based graders highlighting that, in single-shot queries, more comprehensive and educationally rich AI responses are valued over brief ones. This aligns with findings suggesting that LLMs can deliver higher quality and significantly more empathetic responses than human physicians (6). However, this preference for completeness is undermined by a second key finding: The average FKGL for the preferred model, Gemini, was 11.3, with others like Sonar Pro reaching an FKGL of 14.1 (+2.75 grade levels). This is drastically higher than the 6th- to 8th-grade reading level recommended for patient education materials (11, 12). This poor accessibility risks exacerbating health literacy disparities, rendering even the most accurate and complete information useless for a significant portion of the patient population. This aligns with previous findings that noted this is not a problem unique to LLMs; traditional patient education materials often suffer from the same complexity (8, 12). The promise of LLMs lies in their potential for dynamic adjustment, specifically the ability to explicitly adjust output reading level to meet a patient's documented literacy level or preference—a capability that is clearly not a default feature but is a fundamental requirement for equitable deployment.

Furthermore, we noted critical gaps in both contextual awareness and information currency. For instance, when asked about “TASH,” some models failed to disambiguate the acronym within the provided cardiovascular context, offering definitions for “Transurethral Ablation of the Septum of the Heart”. While iterative prompting might resolve this, this limitation highlights the immediate need for more sophisticated, context-aware system instructions to guide model terminology and disambiguation from the first query. In the case of the hallucinated opioid, the chatbot model introduced completely false medical information in a highly confident manner. Such responses may confuse, unnecessarily alarm, or distract patients from their original concern, particularly in vulnerable or high-anxiety contexts. While an expert user might recognize the ambiguity and reformulate the query, this cannot be assumed for all patients, who may interpret the response as meaningful. More broadly, this example highlights a limitation of current medical LLM interactions: when uncertainty exists regarding abbreviations or intent, a safer behaviour would be to request clarification before generating specific medical advice. We identified further failure modes with more direct patient safety implication. These fell into three clinically meaningful categories. The first was potentially harmful recommendation: framing a red-flag symptom cluster for acute cardiac decompensation as a generic differential diagnosis problem and providing management advice that directly contravened guideline-recommended fluid restriction. The second was selective framing: structuring a response around rare but alarming adverse event reports ahead of a clear guideline recommendation, in a manner likely to discourage a vulnerable patient from a clearly beneficial intervention. The third was the omission of a critical diagnostic red flag: failing to recognise bilateral carpal tunnel syndrome as a well-established phenotypic marker of ATTR cardiac amyloidosis - a treatable infiltrative cardiomyopathy whose early identification substantially alters management. Importantly, these failures occurred in responses that were otherwise fluent and internally coherent, and some were generated by models that performed well overall. Also concerning were the content gaps. Models frequently omitted up-to-date management strategies, such as the role of SGLT-2 inhibitors for heart failure, the use of semaglutide for obesity in heart failure, or novel therapy options for LVOT obstruction in HCM. This limitation is consistent with observations by Sarraju et al., who reported that LLM outputs often mirror established consensus while underrepresenting emerging evidence, reflecting the static nature of their training data (9). While web-enabled models aim to solve this by retrieving real-time information, this introduces a trade-off. Their outputs, while potentially more current, can be less controllable and predictable, with source vetting becoming a significant, non-trivial challenge. This lack of currency in static models remains a critical flaw in a field as dynamic as cardiovascular medicine.

Our study builds upon a growing body of literature evaluating LLMs in medicine, but with key distinctions. While earlier studies focused on older models or single platforms, our work provides a contemporary, head-to-head comparison of SOTA competing models (13–15). Hallucinations, or the generation of plausible but false information, is a widely cited concern (10). While our “confabulation avoidance” metric showed strong performance on average, it remains essential to design systems that minimize hallucinations and potentially harmful patient information. This need for caution contrasts with the excellent - and, in some cases, physician-surpassing - diagnostic performance of LLMs in complex cases, suggesting that while their underlying knowledge base is robust, careful and controlled deployment remains essential (7). Since most documented hallucinations involve inaccurate or fabricated references, it is crucial to implement rigorous source validation mechanisms to ensure safety. To assess the confabulation rate of human doctors was beyond the scope of this work, but certainly also needs to be considered in any AI benchmarking approach. Importantly, this evaluation context—patient-facing information delivery—differs fundamentally from the task of guideline-based clinical decision support. Prior assessments of LLMs across the cardiovascular medicine landscape have shown that performance varies substantially with task complexity, with clinical decision-making requiring greatest precision and domain-specific optimization (8, 9). This distinction underscores that the bar for their deployment in clinical reasoning contexts may be considerably higher, reinforcing the need for task-specific validation frameworks.

Our implementation of six automated AI graders for sensitivity analysis introduces a novel dimension to LLM evaluation methodology. This approach directly addresses common limitations in the field, which often relies on small, subjective expert panels, by introducing a scalable, objective, and highly consistent evaluation layer (8). These graders - fully blinded and governed by strict, conservative system instructions - consistently assigned higher scores than humans. While human experts provide irreplaceable clinical nuance, the AI graders provide an additional objective and replicable measure of assessment. Our dual-evaluation methodology, combining expert clinical judgment with scalable AI consistency, thus provides a more robust and comprehensive assessment than either method could achieve alone.

### Implications and future directions

Our findings underscore the need for accessible, reliable tools for the empowered ePatient, who increasingly moves beyond siloed hospital-centric care (16). While general-purpose LLMs show promise, risks regarding readability, contextual errors, and outdated information define critical safety priorities. The “static knowledge” limitation and potential for clinical misguidance can be addressed through Retrieval-Augmented Generation (RAG), grounding responses in real-time, curated guidelines (9, 17). Furthermore, failures in contextual awareness may be mitigated not only by resource-intensive fine-tuning but also by sophisticated system prompting (18, 19). Alternatively, deploying smaller, open-source models locally within hospital infrastructure offers a pathway to ensure data privacy and auditability (20–22). Although these local models may initially demonstrate lower general-purpose performance, they can be highly effective and auditable when optimized for specific, well-defined tasks like patient information on heart failure and cardiomyopathies. Furthermore, all such systems, whether local or cloud-based, should be explicitly engineered for adjustable readability, a necessary step to directly solve the profound health literacy barrier.

Our findings point to two divergent paths for deployment. One immediate, low-risk application is the clinician-in-the-loop model, where the LLM acts as a clinician co-pilot to draft responses, allowing the expert to rapidly customize, verify, and personalize the patient education materials for final vetting. However, this model fundamentally fails to address the primary use case for patient-facing AI: providing information to patients before they consult a clinician, often due to availability or access issues. Therefore, the ultimate goal must be to make direct-to-patient models safe. This requires a new regulatory paradigm where these patient-facing tools are treated as Software as a Medical Device (SaMD), subject to rigorous standards of validation, safety, and post-market surveillance to protect the empowered ePatient (21, 23). Future studies must not only include patients in the evaluation process but also measure the real-world impact on their comprehension, health literacy, and subsequent health behaviors. This patient-centric validation should be applied to the next generation of specialized systems, such as those using RAG or secure, on-premise models, to test their real-world efficacy. Only by validating these tools with patients can we ensure they fulfill their promise of democratizing medical knowledge and supporting a truly patient-centered model of care.

### Strengths and limitations

The primary strength of this study lies in its rigorous, blinded, and randomized evaluation platform. By comparing six leading models on a comprehensive 50-question set, graded by both clinical experts and a panel of AI systems across nine dimensions, we provide a robust and multidimensional snapshot of the current LLM landscape. However, this study has several important limitations. First, our 50-question set cannot capture the full spectrum of patient queries. Second, this static evaluation reflects a specific point in time for rapidly evolving models. Third, the exclusion of direct patient feedback means we may have prioritized completeness over factors patients value more, such as empathy. Fourth, our evaluation rubric did not include a formal severity tier distinguishing no error, minor inaccuracy, and potentially harmful content. Finally, assessing single-shot answers does not reflect the iterative nature of real-world conversational AI interactions.

## Conclusion

This study provides the most comprehensive comparison of leading LLMs for heart failure patient education to date. We demonstrate that while the capabilities of models like Gemini are impressive, significant deficiencies in readability, information currency, and contextual awareness remain among SOTA-LLMs. The potential for LLMs to revolutionize patient education is undeniable, but this potential will only be realized safely through a paradigm of human-in-the-loop supervision. The future of medical LLMs is not as a physician replacement, but as a powerful, critically-evaluated assistant that empowers clinicians to educate their patients more effectively.

## Data Availability

The datasets presented in this study can be found in online repositories. The names of the repository/repositories and accession number(s) can be found below: https://doi.org/10.5281/zenodo.15855267.
