# Supplementary material for: Performance of large language models in delivering accurate and comprehensible patient information on heart failure and cardiomyopathy
Source: Front Digit Health. 2026 Jun 9;8:1847603. doi: 10.3389/fdgth.2026.1847603 (PMC13286976; doi:10.3389/fdgth.2026.1847603)
Supplement: Supplementary file 1 [file Supplementaryfile1.docx]

SUPPLEMENTARY MATERIAL

# Performance of Large Language Models in Delivering Accurate and Comprehensible Patient Information on Heart Failure and Cardiomyopathy

## Supplementary Methods

### API Integration and Query Execution

All LLM responses were collected using a standardized automated pipeline implemented in Python (scripts/run_model_queries.py). Individual API client wrappers were developed for each LLM platform to ensure uniform query handling while accommodating platform-specific requirements. The query script utilized the chatlas package for Claude (Anthropic), Gemini (Google), and Grok (GitHub Models), while custom wrapper classes were implemented for GPT-4o (OpenAI), DeepSeek, Perplexity Sonar Pro, and Grok (X.AI) to maintain consistent response formatting across all models (scripts/utils.py).

### Query Parameters and Execution Protocol

Each of the 50 selected questions was submitted to all six LLMs using their respective API endpoints. To ensure response independence and prevent context carryover between queries, each question was posed as a fresh conversation with no prior message history. All queries were executed with an empty system prompt to simulate raw user access without role-specific priming. Model-specific API versions were selected to represent the most current production releases available at the time of data collection: gpt-4o-2024-11-20 (OpenAI), claude-3-7-sonnet-20250219 (Anthropic), gemini-2.5-pro-preview-05-06 (Google), deepseek-chat (DeepSeek), sonar-pro (Perplexity), and grok-3 (xAI).

### Rate Limiting and Parallel Processing

Queries were executed in parallel using Python's ThreadPoolExecutor with a maximum of 6 concurrent workers to optimize data collection efficiency. Platform-specific rate limiting was implemented to comply with API constraints: Claude requests were serialized with a 1.2-second delay between queries (~50 requests per minute). Automatic retry logic with exponential backoff (minimum 2 seconds, maximum 60 seconds, up to 5 attempts) was implemented for all API calls to handle transient failures.

### Data Storage and Response Processing

All API responses were captured and stored in both structured (CSV) and semi-structured (pickle/JSON) formats with timestamps for reproducibility. The structured output (outputs/chats/responses_TIMESTAMP.csv) contained the response text for each model alongside question metadata (index, category, subcategories, query_id). Raw response objects including usage statistics (token counts) and timestamps were preserved in pickle format (outputs/chats/raw_responses_TIMESTAMP.pkl) with JSON backup for cross-platform compatibility. Model responses were converted directly from markdown except for Sonar Pro, where citations and references were removed during preprocessing to maintain evaluator blinding.

### Automated Evaluation by AI-Based Graders

To complement human evaluations, we implemented a structured AI-based grading system using function calling with publicly accessible large language models (LLMs). Six AI graders were configured using GPT-4o, Gemini 2.0 Flash, Gemini 2.5 Pro, Claude 3.5 Sonnet, and Claude 4. Each grader was deployed via API with standardized prompts and structured output formatting. All AI graders were assigned the same system prompt, which instructed them to act as strict, professional grading assistants tasked with evaluating chatbot responses to patient-facing medical questions. The prompt emphasized conservative scoring, objective judgment, and a focus on clinical quality and patient communication. Specifically, graders were told:

You are a strict, professional grading assistant responsible for evaluating chatbot responses to medical questions posed by patients. Your primary goal is to help improve the quality of these answers making them clearer, more useful, and more empathetic for patient understanding and decision-making.

You will assess each response across multiple quality dimensions using a Likert scale from 1 to 5:

1 = Very poor

2 = Poor

3 = Neutral or Average

4 = Very Good

5 = Outstanding

Grade conservatively: only assign a 5 when the response is outstanding. Base your evaluation on clinical appropriateness, factual correctness, and patient communication quality.Be objective, strict and consistent in your judgments. Avoid inflating scores or giving top scores if the answer is not outstanding. Strictly grade. Be conservative: a typical correct answer should score a 3 or 4, not a 5.

Each model response was evaluated across nine predefined quality dimensions: Appropriateness, Comprehensibility, Completeness, Conciseness, Confabulation Avoidance, Readability, Educational Value, Actionability, and Tone/Empathy. Graders also recorded a categorical trust judgment in response to: “Would you feel comfortable if a patient relied on this answer?” with choices: “Yes, fully comfortable,” “Somewhat comfortable,” and “Not comfortable.”

Function calling or equivalent structured output tools were used to elicit model-generated scores for each criterion. To reduce stochasticity, temperature was fixed at 0.1, and retry logic was implemented to handle rate limiting. Model outputs were parsed automatically and merged with the human evaluation dataset for sensitivity and comparative analyses. All grading prompts, structure specifications, and implementation code are available at: <https://github.com/q050cr/llm_grading_app>.

### Post-hoc same-company grader bias analysis

To assess whether auto-graders scored their own company's model more favourably than other models, we conducted a two-step analysis using the individual rating records. Auto-grader families were defined by provider: OpenAI (GPT-4o, GPT-4o-strict), Anthropic (Claude 3.5 Sonnet, Claude 3.7 Sonnet), and Google (Gemini 2.0 Flash, Gemini 2.5 Pro). For each family, the own-company model was GPT-4o, Claude 3.7 Sonnet, and Gemini 2.5 Pro, respectively.

**Step 1 Raw score comparison.** For each auto-grader family, we computed the mean score assigned to the own-company model and the mean score assigned to each of the five other models across all questions and rating features (9 dimensions, 50 questions). The same-company bias estimate was defined as the difference: mean score own − mean score others. Bootstrap 95 % confidence intervals were obtained by resampling question × feature cells with replacement within each grader family (n = 5,000 resamples).

**Step 2 Ceiling correction.** On a bounded 1–5 scale, models that already receive high human-consensus scores have less remaining headroom for further inflation, which can artificially suppress the apparent same-company effect for high-quality models and inflate it for lower-quality ones. We therefore computed, for each grader family and each model, the available headroom (5.0 − human rater mean) and the grader delta (auto-grader mean − human rater mean). A simple linear regression of delta on headroom was fitted using only the five non-own models (45 model × feature data points per grader family). The ceiling-corrected same-company bias was then defined as the actual delta for the own model minus the delta predicted by that regression — i.e., the residual relative to the grader's general leniency pattern. Bootstrap 95 % confidence intervals were obtained by resampling model × feature cells within each grader family (n = 5,000 resamples).

## Supplementary Tables

### Supplement Table S1: Readability Metrics by Category and Model.

| **Modell** | **Flesch Ease** | **Flesch K-G** | **Gunning Fog** | **SMOG** | **Coleman-Liau** | **ARI** |
| --- | --- | --- | --- | --- | --- | --- |
| **Disease Understanding and Diagnosis** | | | | | | |
| Claude | 30.0 ± 16.1 | 11.7 ± 2.2 | 15.4 ± 2.7 | 12.8 ± 1.3 | 16.4 ± 2.6 | 11.9 ± 2.1 |
| DeepSeek | 34.0 ± 14.0 | 11.5 ± 2.1 | 14.7 ± 2.7 | 12.8 ± 1.5 | 15.4 ± 2.4 | 11.6 ± 2.2 |
| Gemini | 40.7 ± 17.1 | 11.3 ± 2.6 | 14.6 ± 3.2 | 13.3 ± 2.1 | 13.9 ± 2.8 | 11.5 ± 2.6 |
| GPT-4o | 35.9 ± 13.6 | 12.1 ± 2.3 | 15.7 ± 3.0 | 14.0 ± 2.0 | 14.8 ± 2.1 | 12.4 ± 2.3 |
| Sonar Pro | 32.7 ± 14.9 | 13.6 ± 2.6 | 17.3 ± 3.3 | 15.1 ± 2.2 | 14.7 ± 2.3 | 14.2 ± 2.8 |
| Grok | 37.4 ± 12.9 | 12.2 ± 1.9 | 15.6 ± 2.6 | 14.0 ± 1.7 | 14.3 ± 1.9 | 12.4 ± 1.8 |
| **Treatment and Management** | | | | | | |
| Claude | 22.8 ± 11.3 | 12.6 ± 1.7 | 16.4 ± 2.2 | 13.2 ± 1.3 | 17.6 ± 2.1 | 12.8 ± 1.8 |
| DeepSeek | 32.5 ± 9.9 | 12.0 ± 1.6 | 15.5 ± 2.2 | 13.5 ± 1.5 | 15.3 ± 1.4 | 12.0 ± 1.6 |
| Gemini | 38.1 ± 8.8 | 11.5 ± 1.4 | 15.0 ± 1.8 | 13.5 ± 1.1 | 14.2 ± 1.6 | 11.4 ± 1.6 |
| GPT-4o | 31.9 ± 7.4 | 13.0 ± 1.5 | 16.7 ± 1.7 | 14.7 ± 1.3 | 15.5 ± 1.3 | 13.5 ± 1.9 |
| Sonar Pro | 24.6 ± 9.8 | 14.5 ± 1.5 | 18.3 ± 1.8 | 15.8 ± 1.2 | 16.7 ± 1.7 | 15.3 ± 1.6 |
| Grok | 34.9 ± 6.6 | 13.4 ± 1.2 | 16.9 ± 1.8 | 15.1 ± 1.3 | 14.4 ± 1.0 | 14.1 ± 1.3 |
| **Lifestyle & Daily Activity** | | | | | | |
| Claude | 26.7 ± 14.0 | 11.8 ± 2.0 | 15.6 ± 3.0 | 12.4 ± 1.4 | 16.7 ± 2.5 | 11.8 ± 2.1 |
| DeepSeek | 34.7 ± 9.6 | 11.4 ± 1.6 | 14.8 ± 2.6 | 12.9 ± 1.5 | 14.9 ± 1.8 | 11.2 ± 1.9 |
| Gemini | 41.1 ± 12.5 | 10.7 ± 2.2 | 14.0 ± 2.6 | 12.8 ± 1.7 | 13.5 ± 2.0 | 10.4 ± 2.2 |
| GPT-4o | 36.0 ± 10.7 | 12.4 ± 1.6 | 16.2 ± 2.4 | 14.4 ± 1.6 | 14.4 ± 1.6 | 12.6 ± 1.5 |
| Sonar Pro | 30.2 ± 12.4 | 13.5 ± 2.0 | 17.3 ± 2.7 | 15.2 ± 1.8 | 15.7 ± 1.5 | 14.1 ± 1.7 |
| Grok | 35.9 ± 9.3 | 12.6 ± 1.8 | 16.0 ± 2.6 | 14.4 ± 1.8 | 14.2 ± 1.4 | 12.8 ± 2.0 |

**Supplement Table S2: Word Count by Model**

| **Model** | **Word Count** |
| --- | --- |
| Grok | 671.2 ± 202.3 |
| Gemini | 668.7 ± 116.1 |
| GPT-4o | 475.8 ± 133.4 |
| Sonar Pro | 346.4 ± 63.4 |
| DeepSeek | 299.5 ± 57.8 |
| Claude | 226.9 ± 38.9 |

### Supplement Table S3: Reasons for Model Preference

| **Reason** | **Count** | **Percentage (%)** |
| --- | --- | --- |
| **Claude** | | |
| Easier to understand (lay language) | 8 | 36.4 |
| Best reflects clinical practice | 7 | 31.8 |
| Clearer explanation | 3 | 13.6 |
| More empathetic tone | 2 | 9.1 |
| Safest advice | 2 | 9.1 |
| **DeepSeek** | | |
| Clearer explanation | 3 | 33.3 |
| Easier to understand (lay language) | 2 | 22.2 |
| Safest advice | 2 | 22.2 |
| Best reflects clinical practice | 1 | 11.1 |
| More complete content | 1 | 11.1 |
| **Gemini** | | |
| Clearer explanation | 46 | 35.1 |
| More complete content | 41 | 31.3 |
| Best reflects clinical practice | 18 | 13.7 |
| Safest advice | 11 | 8.4 |
| Easier to understand (lay language) | 9 | 6.9 |
| More empathetic tone | 6 | 4.6 |
| **GPT-4o** | | |
| Easier to understand (lay language) | 11 | 31.4 |
| Clearer explanation | 9 | 25.7 |
| More empathetic tone | 5 | 14.3 |
| Safest advice | 4 | 11.4 |
| Best reflects clinical practice | 3 | 8.6 |
| More complete content | 3 | 8.6 |
| **Sonar Pro** | | |
| Easier to understand (lay language) | 4 | 33.3 |
| Best reflects clinical practice | 2 | 16.7 |
| Clearer explanation | 2 | 16.7 |
| More complete content | 2 | 16.7 |
| More empathetic tone | 1 | 8.3 |
| Safest advice | 1 | 8.3 |
| **Grok** | | |
| Easier to understand (lay language) | 29 | 31.9 |
| Clearer explanation | 24 | 26.4 |
| More complete content | 19 | 20.9 |
| Best reflects clinical practice | 7 | 7.7 |
| Safest advice | 7 | 7.7 |
| More empathetic tone | 5 | 5.5 |

### Supplement Table S4: Rater Model Preference

| **Preferred Model** | **Count** | **Percentage (%)** |
| --- | --- | --- |
| Expert 1 | | |
| Gemini | 16 | 32 |
| Claude | 12 | 24 |
| Grok | 8 | 16 |
| GPT-4o | 7 | 14 |
| Sonar Pro | 5 | 10 |
| DeepSeek | 2 | 4 |
| Expert 2 | | |
| Gemini | 31 | 62 |
| Grok | 17 | 34 |
| DeepSeek | 2 | 4 |
| Expert 3 | | |
| Grok | 23 | 46 |
| GPT-4o | 13 | 26 |
| Claude | 6 | 12 |
| DeepSeek | 4 | 8 |
| Gemini | 3 | 6 |
| Sonar Pro | 1 | 2 |
| Student 1 | | |
| Gemini | 21 | 42 |
| Grok | 19 | 38 |
| GPT-4o | 4 | 8 |
| Sonar Pro | 4 | 8 |
| Claude | 2 | 4 |
| Student 2 | | |
| Gemini | 29 | 58 |
| Grok | 13 | 26 |
| GPT-4o | 7 | 14 |
| Claude | 1 | 2 |
| Student 3 | | |
| Gemini | 31 | 62 |
| Grok | 11 | 22 |
| GPT-4o | 4 | 8 |
| Sonar Pro | 2 | 4 |
| Claude | 1 | 2 |
| DeepSeek | 1 | 2 |

### Supplement Table S4: Post-hoc same-company auto-grader bias analysis

*Raw difference = mean score given to own-company model minus mean score given to the five other models (bootstrap 95 % CI, n = 5,000 resamples; 1–5 Likert scale). Ceiling-corrected bias = residual after accounting for available headroom on a bounded scale (linear regression of auto-grader − human-rater delta on headroom fitted on five non-own models, applied to own model; bootstrap 95 % CI by resampling model × feature cells, n = 5,000). No grader shows a statistically significant own-company effect after ceiling correction (all 95 % CIs cross zero). CI, confidence interval.*

| **Own model** | **Mean score own** | **Mean scores others** | **Raw difference (95 % CI)** | **Ceiling-corrected bias (95 % CI)** |
| --- | --- | --- | --- | --- |
| GPT-4o | 4.47 | 4.48 | −0.01 (−0.06 to +0.03) | −0.02 (−0.30 to +0.20) |
| Claude | 4.62 | 4.55 | +0.07 (+0.03 to +0.11) | +0.19 (−0.12 to +0.47) |
| Gemini | 4.84 | 4.66 | +0.18 (+0.15 to +0.21) | +0.01 (−0.21 to +0.22) |
